# Nrf2 activation drive macrophages polarization and cancer cell epithelial-mesenchymal transition during interaction

**DOI:** 10.1186/s12964-018-0262-x

**Published:** 2018-09-04

**Authors:** Rui Feng, Yuji Morine, Tetsuya Ikemoto, Satoru Imura, Shuichi Iwahashi, Yu Saito, Mitsuo Shimada

**Affiliations:** Department of Surgery, Institute of Biomedical Sciences, Tokushima University of Graduate School, 3-18-15 Kuramoto-cho, Tokushima, 770-8503 Japan

**Keywords:** Nuclear factor (erythroid-derived 2)-like 2, Tumor-associated macrophage, Hepatocellular carcinoma, Pancreatic cancer, Epithelial-mesenchymal transition

## Abstract

**Background:**

The M2 phenotype of tumor-associated macrophages (TAM) inhibits the anti-tumor inflammation, increases angiogenesis and promotes tumor progression. The transcription factor Nuclear Factor (erythroid-derived 2)-Like 2 (Nrf2) not only modulates the angiogenesis but also plays the anti-inflammatory role through inhibiting pro-inflammatory cytokines expression; however, the role of Nrf2 in the cancer cell and macrophages interaction is not clear.

**Methods:**

Hepatocellular carcinoma cells (Hep G2 and Huh 7) and pancreatic cancer cells (SUIT2 and Panc-1) were co-cultured with monocytes cells (THP-1) or peripheral blood monocytes derived macrophages, then the phenotype changes of macrophages and epithelial-mesenchymal transition of cancer cells were detected. Also, the role of Nrf2 in cancer cells and macrophages interaction were investigated.

**Results:**

In this study, we found that cancer cells could induce an M2-like macrophage characterized by up-regulation of CD163 and Arg1, and down-regulation of IL-1b and IL-6 through Nrf2 activation. Also, Nrf2 activation of macrophages promoted VEGF expression. The Nrf2 activation of macrophages correlated with the reactive oxygen species induced by cancer cells derived lactate. Cancer cells educated macrophages could activate Nrf2 of the cancer cells, in turn, to increase cancer cells epithelial-mesenchymal transition (EMT) through paracrine VEGF. These findings suggested that Nrf2 played the important role in the cancer cells and macrophages interaction.

**Conclusions:**

Macrophage Nrf2 activation by cancer cell-derived lactate skews macrophages polarization towards an M2-like phenotype and educated macrophages activate Nrf2 of the cancer cells to promote EMT of cancer cells. This study provides a new understanding of the role of Nrf2 in the cancer cell and TAM interaction and suggests a potential therapeutic target.

**Electronic supplementary material:**

The online version of this article (10.1186/s12964-018-0262-x) contains supplementary material, which is available to authorized users.

## Background

The tumor microenvironment, mainly consisted of extracellular matrix, fibroblasts, vascular endothelial cells and immunocytes, plays an important role in tumor structure and biology, contributing tumor cells survival, proliferation and migration [1]. Macrophages are attracted and activated by tumor microenvironment derived cytokines, differentiating into tumor-associated macrophages (TAMs) and contributing to complicating inflammation conditions [2]. It has indicated that TAMs were multifunctional and heterogeneous [3]. The one phenotype of TAMs shows the pro-inflammatory characteristic is identified as classical or M1 macrophages, which secret IL-1 and IL-6; the other phenotype owns anti-inflammatory capability referred to alternative or M2 macrophages, which mainly express Arg1 and CD163 [4]. The current studies have shown that higher amount of M2 TAMs was associated with worse clinical prognosis [5–7]. A higher density of M2 TAMs appears to correlate with tumor cell proliferation, metastasis, and intra-tumoral microvascular density [8, 9].

Nuclear factor (erythroid-derived 2)-like 2 (Nrf2), which is essential for keeping the balance of oxidative stress, has been known to improve cell survival under threaten of xenobiotic toxicants through initiating anti-oxidative genes transcription [10]. Without stimulation, Nrf2 binds with two molecules of Keap-1 and is subsequently proteasomal degraded. Oxidative stressors or electrophiles dissociates the Nrf2 from Keap-1, prevents Nrf2 from degrading and increases nuclear accumulation of Nrf2. Also, Nrf2 has been shown to favor the progression, chemo- and radio-resistance of several types of cancers [11–13]. Previous studies have demonstrated that Nrf2 could directly inhibit the inflammatory cytokines transcription of immune cells and indirectly limit the inflammatory process through modulating HO-1 expression [14, 15]. However, the role of Nrf2 in TAM formation and its function on cancer cell is unclear.

In this study, THP-1 or peripheral blood monocyte (PBMC) derived macrophages and cancer cells co-stimulation was performed to mimic the cancer cell and macrophages interaction in the tumor microenvironment. We found that Nrf2 was essential for driving macrophage activation into M2 phenotype by cancer cell stimulation and migration of cancer cell.

## Methods

### Cell culture and treatment

The hepatocellular carcinoma cell lines Hep G2 and Huh 7 were gotten from the Riken Cell Bank (Tsukuba, Japan). Pancreatic cancer cell lines Panc-1 was gotten from the Riken Cell Bank, and SUIT2 was gotten from Japanese Collection of Research Bio-resources Cell Bank (Ibaraki, Japan). These cell lines were passaged for fewer than 6 months after resuscitation. Cancer cells were cultured in DMEM (Life Technologies Japan Ltd., Tokyo, Japan) containing 10% FBS (Life Technologies Japan Ltd., Tokyo, Japan). Human monocyte cell line, THP-1, gotten from Culture Collections of Public Health England, grew in RPMI-1640 (Wako, Osaka, Japan) containing 10%FBS and 10 mM HEPES. THP-1 cells were treated with 150 nM phorbol-12-myristate-13-acetate (PMA) (Sigma, St. Louis, MO, USA) for 48 h to induce to macrophages.

Cells were transfected with Nrf2 siRNA (Applied Biosystems, s9492, Waltham, Massachusetts, USA), Keap-1 siRNA (Applied Biosystems, s18981, Waltham, MA, USA) or negative control siRNA (Applied Biosystems, Select Negative Control #1 siRNA, Waltham, MA, USA) at 10 nmol/L using Lipofectamine 2000 (Invitrogen, Thermo Fisher Scientific Inc., Waltham, MA, USA) according to the manufacturer’s instructions.

### Conditioned medium (CM) of cells preparing

The cells were washed with pre-warmed PBS twice, then incubated with the FBS-free medium. After 24 h of incubation, the medium was collected, centrifuged (450×g, 5 mins) and filtrated by the 0.2 μm filter. The conditioned medium was used without additional FBS supplemented.

### Migration assay

The transwell inserts (Corning, NY. USA) with 8 μm pore size were used for migration assay. 0.5-1 × 10^5^ cancer cells were seeded in the upper chamber. After cell attached, the upper chambers were washed twice with DMEM gently, then the medium of upper chambers was changed to conditioned medium, and the same medium with 5% FBS supplemented was used in the lower chamber. After 24 h incubation, the cells on the downside of transwell inserts were fixed in 4% paraformaldehyde and stained with 0.2% crystal violet. The stained cells in five random × 400 microscopic fields were counted.

### Lactate assay

The concentration of lactate in the conditioned medium was measured by using lactate assay kit (Sigma, St. Louis, MO, USA). The 10ul fresh conditioned medium was mixed with lactate assay buffer. Then 50ul of the master reaction mix was added to each sample. After incubating the reaction for 30 min at room temperature, measure the absorbance at 570 nm.

### Reactive oxygen species (ROS) detection

The cells were incubated with 2′, 7′-Dichlorofluorescin diacetate (H2DCFDA 5 μM, Sigma) 30 min. Then the cells were washed and received the stimulation of conditioned medium for 6 h. The fluorescence intensity (λex = 495/λem = 529 nm) was measured by the plate reader (SpectraMax i3, Molecular Devices, Tokyo, Japan).

### Enzyme-linked immunosorbent assay

The level of IL-1, IL-6 and VEGF were detected by using IL-1, IL-6 and VEGF Quantikine ELISA kit (R&D Systems, Minneapolis, MN, USA) according to the manufacturer’s protocol. Absorbance at 450 nm was measured using a plate reader as a correction wavelength of 540 nm.

### Polymerase chain reaction

The total RNA of each sample was extracted using the RNeasy Mini Kit (Qiagen, Hilden, Germany) according to the manufacturer’s instructions. cDNA was synthesized using the reverse transcription kit (Applied Biosystems, Thermo Fisher Scientific Inc., Waltham, MA, USA). The following primers from TaqMan gene expression assays (assay identification number) were used: IL-1b (Hs01555410_m1); IL-6 (Hs00174131_m1); CD163 (Hs00174705_m1); Arg1 (Hs00163660_m1) and VEGF-α (Hs00163660_m1). GAPDH (4352339E) was selected as internal control. StepOnePlus Real-Time PCR System (Applied Biosystems, Thermo Fisher Scientific Inc., Waltham, MA, USA) was used to perform qRT-PCR.

### Western blotting

Nuclear and cytoplasmic proteins were extracted by using NuCLEAR™ Extraction Kit (Sigma, St. Louis, MO, USA). Total proteins were extracted by lysing cell with RIPA buffer (Thermo Fisher Scientific Inc., Waltham, MA, USA) containing the protease inhibitor cocktail (Sigma, St. Louis, MO, USA) and PhosSTOP phosphatase inhibitor cocktail (Roche, Tokyo, Japan). Then Proteins concentration were measured with the BCA kit (Thermo Fisher Scientific Inc., Waltham, MA, USA), and equal amounts of extracted proteins separated on 10% SDS-PAGE gels and transferred onto PVDF membranes (Bio-Rad, Hercules, CA, USA). Membranes were incubated with the indicated primary antibody, then appropriate HRP-conjugated secondary antibody. The proteins were detected with Chemiluminescence (GE, Little Chalfont, Buckinghamshire, UK). The primary antibodies used in western blotting were listed in Table 1.

**Table 1:** The first antibody used in western blot

Name	Product number	Company
Histone H3 antibody	#9715	Cell Signaling
b-Actin antibody	#4967	Cell Signaling
N-cadherin antibody	#13116	Cell Signaling
Nrf2 antibody	ab62352	Abcam
E-cadherin antibody	ab1416	Abcam

### Tumor cell transplantation

Six to eight-week-old BALB/c nude mice were purchased from Charles River Company (Yokohama, Japan) and housed at the animal center of Tokushima University. All animal experiments were approved by Tokushima University Institutional Review Board. The experiments performed in accordance with the guidelines of the Animal Research Committee of Tokushima University Graduate School. To establish the animal model, 250ul of Panc-1 and Huh 7 (2 × 10^7^ cells/ml) containing 50% matrigel matrix (Corning, NY. USA) with or without educated macrophages (2 × 10^5^ cells) was injected subcutaneously. After four weeks, the mice were sacrificed and tumor tissues were collected.

### Immunochemistry

The cells were washed with cold PBS and fixed with paraformaldehyde. Then the fixed cells were incubated with anti-Nrf2 primary antibody overnight at 4 °C. Then after fluorophore-conjugated second antibody incubation, the cells were observed. The xenograft tumor tissues, which paraformaldehyde –fixed and paraffin-embedded, were incubated with primary monoclonal antibody CD163 (ab87099, Abcam, Tokyo, Japan) and Nrf2 (ab62352, Abcam, Tokyo, Japan). After incubated with the fluorophore-conjected second antibody, the slides were checked by fluorescence microscope (Keyence Corporation, Itasca, IL, USA).

### Statistical analysis

Data were presented as mean ± SD. Statistical analyses were performed by SPSS 19.0 (IBM, NY, USA). The student t-test was used to compare the difference between the two groups. One-way analysis of variance (one-way ANOVA) followed by Bonferroni’ test was used to compare the differences more than two groups. There was a statistically significant difference when the *p*-value was less than 0.05.

## Results

### Cancer cells induced macrophages phenotype transformation

**Fig. 1:**
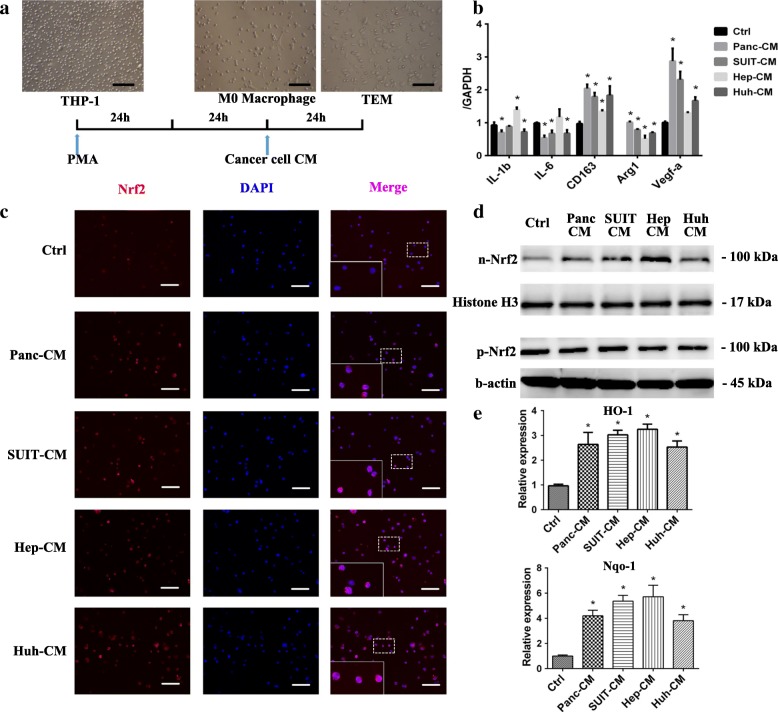
Cancer cells induced macrophages phenotype transformation and Nrf2 activation. **a** THP-1 cells were incubated with PMA (150 nM) 48 h to induced macrophage and then stimulated with cancer cell CM (scale bar 200 μm). **b** after 24 h stimulation with the CM of Panc-1 (Panc-CM), SUIT 2 (SUIT-CM), Hep G2 (Hep-CM) and Huh 7 (Huh-CM) or cultured with normal medium (Ctrl), the genes expressions of macrophages were detected by PCR analysis (*n* = 4). **c**, after 24 h stimulation with the CM of Panc-1 (Panc-CM), SUIT 2 (SUIT-CM), Hep G2 (Hep-CM) and Huh 7 (Huh-CM) or cultured with normal medium (Ctrl), Nrf2 of TEMs and M0 macrophage (red) was stained (scale bar 100 μm) (*n* = 3). **d** the nuclear and cytoplasm Nrf2 of TEM were detected by western blot. **e** the expression of downstream genes of Nrf2 (HO-1 and Nqo-1) were analyzed by PCR analysis (*n* = 4). Graphs show the data as mean ± SD. *, *P* < 0.05, compared with ctrl

After stimulated with PMA 48 h, the suspended THP-1 attached the dishes and differentiated into macrophages (M0) [16]. Then the M0 macrophages were stimulated with CM of hepatocellular carcinoma cells (Hep G2 and Huh 7) and pancreatic carcinoma cells (Panc-1 and SUIT2) for another 24 h (Fig. 1a). After CM stimulation, the tumor educated macrophages (TEM) decreased the expression of M1 macrophage markers, such as IL-1b, IL-6 and increased the expression of M2 macrophage markers including Arg1 and CD163 compared with M0 macrophage. Also, the VEGF-α was up-regulated after education (Fig. 1b). As the same as THP-1 derived macrophages, the human peripheral blood monocyte (PBMC) derived macrophages increased M2 macrophage markers and VEGF expression after CM of cancer cells stimulation (Additional file 1: Figure S1A).

### Nrf2 activation correlated with M2 TEM formation and VEGF-α expression

It has been reported that Nrf2 played the important role in the anti-inflammatory action [17]. Over-activation of Nrf2 could inhibit the pro-inflammatory cytokines expression [15]. In order to uncover whether the Nrf2 participated in the TEM phenotype transformation, Nrf2 expression of TEM was detected firstly. The immunofluorescent staining of Nrf2 demonstrated that the Nrf2 nuclear translocation of TEM apparently increased compared with M0 macrophages (Fig. 1c). Also, compared with the nuclear Nrf2 of M0, nuclear Nrf2 of TEM had an obviously higher level (Fig. 1d). HO-1 and Nqo-1, as the downstream genes of Nrf2, increased after cancer cell CM stimulation (Fig. 1e). The Nrf2 levels were also detected in PBMC derived TEM. Similar to THP-1 derived TEM, PBMC derived TEM also showed an increase of Nrf2 nuclear translocation (Additional file 1: Figure S1B-C). The xenograft tumor tissues of cancer cells and THP-1 derived TEM co-injection and patients’ tumor tissues showed that CD163-positive cells were also characterized as high Nrf2 nuclear-translocation (Additional file 1: Figure S1D-E). Above all, it has been demonstrated that Nrf2 was over-activated in the TEM by cancer cell CM stimulation.

**Fig. 2:**
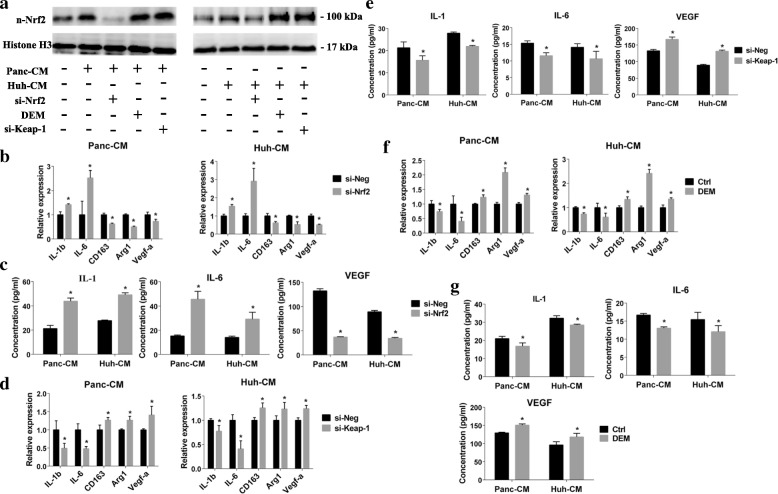
Nrf2 activation was correlated with M2 TEM formation and VEGF-α expression. **a** the siRNA of Nrf2 and Keap-1 and DEM were used to pre-modulate Nrf2 activation. The nuclear Nrf2 were detected by western blotting. **b** the M1/2 macrophages markers of Nrf2 knocked-down (si-Nrf2) were detected after cancer cell CM stimulation (*n* = 4). **c** 24-h secretion of IL-1, IL-6 and VEGF of Nrf2 knocked-down TEM were evaluated by ELISA (*n* = 3). **d** the M1/2 macrophages markers of Keap-1 knocked-down (si-Keap-1) were detected after cancer cell CM stimulation (*n* = 4). **e** IL-1, IL-6 and VEGF concentration of Keap-1 knocked-down (si-Keap-1) TEM were evaluated by ELISA (*n* = 3). **f** M0 macrophages were pre-treated with 100-mM DEM for 6 h before cancer cell CM stimulation, the M1/2 macrophages markers were detected by PCR (*n* = 4). **g**, IL-1, IL-6 and VEGF concentration of DEM pre-treated (DEM) were evaluated by ELISA (*n* = 3). Graphs show the data as mean ± SD. *, *P* < 0.05, compared with si-Neg or ctrl

To confirm the correlation between Nrf2 activation and TEM phenotype transformation, Nrf2 knocked-down of THP-1 derived M0 macrophage was performed by small interfering RNA treatment (Fig. 2a). The Nrf2 knocked-down was also verified by Nrf2 downstream genes expression (Additional file 1: Figure S2A). Nrf2 knock-down of macrophage canceled the effect of cancer cell CM which induced M1 macrophage markers down-regulation and M2 macrophage markers up-regulation (Fig. 2b-c and Additional file 1: Figure S2B). Moreover, Nrf2 knock-down diminished the cancer cell CM-induced VEGF-α expression and secretion (Fig. 2b-c).

Keap-1 mediated Nrf2 degradation is the dominating pathway to modulate Nrf2 activation [18]. When the cell receives stimulation, Nrf2-Keap-1 complex disassembles and Nrf2 translocates into nuclear. Keap-1 knocked-down was utilized to manipulate the Nrf2 activation. Increased Nrf2 nuclear accumulation and Nrf2-dependent transcription were confirmed after Keap-1 knocked-down (Fig. 2a and Additional file 1: Figure S2A). Keap-1 knocked-down amplified M2-macrophages induction by cancer cells, appearing as decreased IL-1b and IL-6 expression, and increased CD163 and Arg1 expression (Fig. 2d-e and Additional file 1: Figure S2B). The application of Diethyl maleate (DEM), a small molecule Nrf2 activator, was also confirmed that Nrf2 activation of macrophages enhanced cancer cell-induced M2 macrophages transformation and VEGF-α expression [15] (Fig. 2f-g and Additional file 1: Figure S2B).

### Lactate secreted by cancer cell promoted TEM Nrf2 activation

**Fig. 3:**
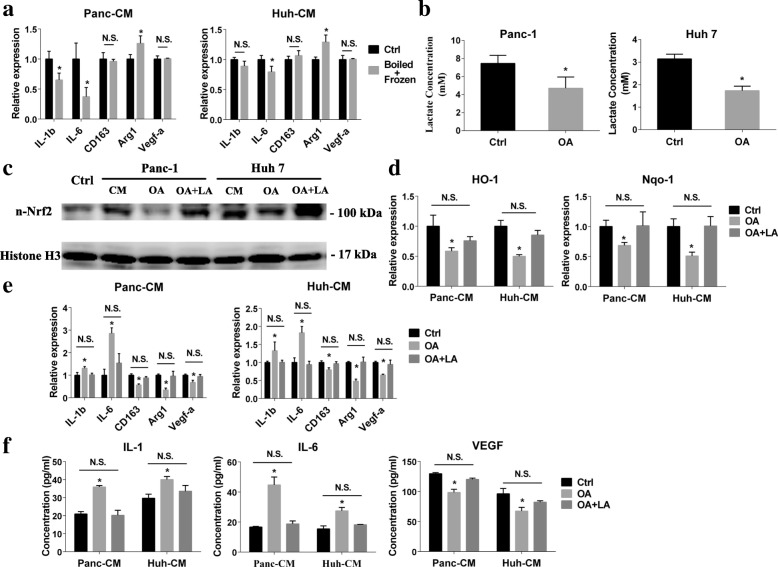
Lactate derived from cancer cells induced macrophages transformation through regulating Nrf2 activation. **a** macrophages were stimulated with cancer cell CM which subjected to three freeze-thaw cycles (− 80 °C, 60 °C), and the expressions of M1/2 macrophages markers were detected by PCR (*n* = 4). **b** cancer cells were incubated with oxalic acid (OA) (30 mM) 3 days before conditioned medium prepared. The lactate concentrations of cancer cell CM were measured (*n* = 3). **c** nuclear Nrf2 of macrophages were detected after cultured with normal medium (Ctrl) or stimulated with the normal cancer cell CM (CM), oxalic acid pre-treated cancer cell CM (OA), or exogenous lactate supplement contained oxalic acid pre-treated cancer cell CM (OA + LA). **d** HO-1 and Nqo-1 expression of TEM after stimulated with the normal cancer cell CM (Ctrl), oxalic acid pre-treated cancer cell CM (OA), or exogenous lactate supplement contained oxalic acid pre-treated cancer cell CM (OA + LA) were analyzed by PCR (*n* = 4). **e** M1/2 macrophage markers expression of TEM after stimulated with the normal cancer cell CM (Ctrl), oxalic acid pre-treated cancer cell CM (OA), or exogenous lactate supplement contained oxalic acid pre-treated cancer cell CM (OA + LA) were detected by PCR (*n* = 4). **f** 24-h secretion of IL-1, IL-6 and VEGF of TEM stimulated with the normal cancer cell CM (Ctrl), oxalic acid pre-treated cancer cell CM (OA), and exogenous lactate supplement contained oxalic acid pre-treated cancer cell CM (OA + LA) were measured by ELISA (*n* = 3). Graphs show the data as mean ± SD. *, *P* < 0.05, compared with ctrl. N.S., no significant between two groups

Our data revealed that the cancer cell CM activated Nrf2 in macrophage correlated with M2 features of TEM, we next sought to identify the tumor-derived soluble factor(s) which was capable of Nrf2 activation. Firstly, to determine the nature of cancer cell secreted factors that alter macrophage subtype, the cancer cell conditioned medium was subjected to three freeze-thaw cycles (− 80 °C, 60 °C). We found that CM of the cancer cell with or without processing both could increase M2 feature of TEM indicating that the factor(s) lacked tertiary structure (Fig. 3a). It meant that proteins which achieved functions relying on the tertiary and quaternary structure were excluded [19]. So, small molecule metabolites could be the candidates. The well-known Warburg effect demonstrates that lactate would be highly produced by cancer cell through aerobic glycolysis even at normoxia condition [20]. Oxalic acid was the one of LDH inhibitor which converted pyruvate to lactate [21]. To verify whether lactate in cancer cell CM correlated with the Nrf2 activation of TEM, we reduced the lactate in cancer cell CM by pre-incubated the cancer cell with the oxalic acid containing media (Fig. 3b). It showed that the lactate-reduced CM of cancer cells lost the capability of promoting Nrf2 activation of macrophages which could be rescued by exogenous lactate supplement (3 mM) (Fig. 3c-d). However, the exogenous hydrochloric acid which could also decrease the pH value as lactate could not promote the Nrf2 activation of macrophages (**Data were not showed**). Concomitantly, lactate-reduced CM of cancer cell failed to induce TEM phenotype transformation until exogenous lactate added (Fig. 3e-f and Additional file 1: Figure S3A-B).

### ROS formation regulated by lactate activated Nrf2

**Fig. 4:**
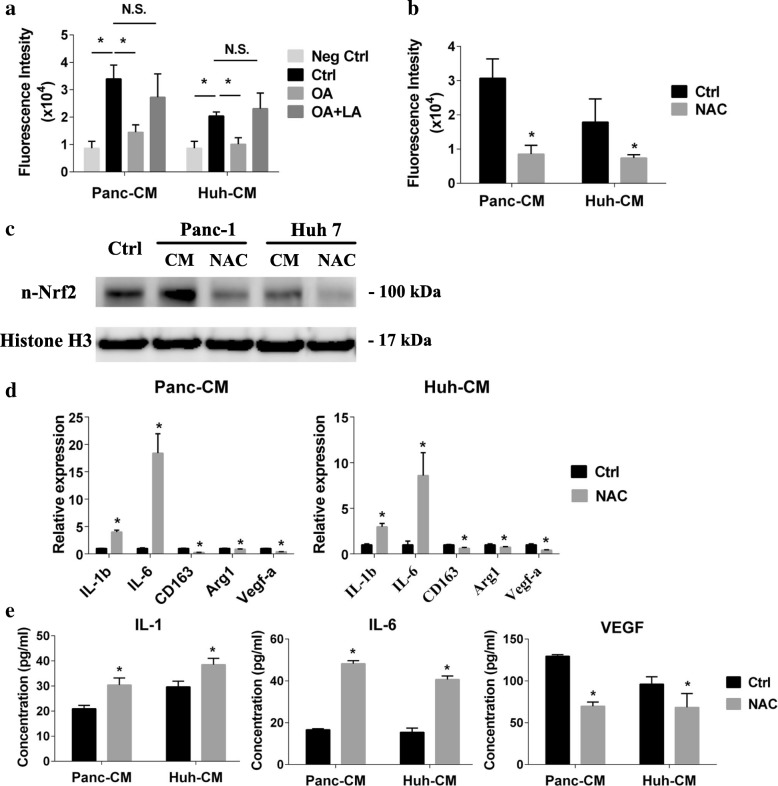
Lactate elevated intracellular ROS of macrophages to induce M2 TEM formation through Nrf2. **a** macrophages were incubated with 2′,7′-dichlorodihydrofluorescein diacetate (H2DCFDA) for 30 mins, then stimulated with FBS-free medium (Neg Ctrl), the normal cancer cell CM (Ctrl), oxalic acid pre-treated cancer cell CM (OA), or exogenous lactate supplement contained oxalic acid pre-treated cancer cell CM (OA + LA) for another 6 h, the fluorescent intensity of 2′,7′-dichlorofluorescein (DCF) were detected (*n* = 4). **b** macrophages were incubated with 2′,7′-dichlorodihydrofluorescein diacetate (H2DCFDA) for 30 mins, then stimulated with normal cancer cell CM with (NAC) or without NAC (Ctrl). The fluorescent intensity of 2′,7′-dichlorofluorescein (DCF) was detected (*n* = 4). **c** the nuclear Nrf2 of macrophages after stimulation by cancer cell CM with or without NAC was measured by western blot. **d** M1/2 macrophages markers expression of TEM induced by cancer cell CM or cancer cell CM and NAC (*n* = 4). **e** 24-h secretion of IL-1, IL-6 and VEGF of macrophages stimulated by cancer cell CM with or without NAC (*n* = 3). Graphs show the data as mean ± SD. *, *P* < 0.05, compared between two groups or with ctrl. N.S., no significant between two groups

In the previous results, we confirmed that the lactate derived from the cancer cell activated the Nrf2 of TEM and induced its M2 feature. However, the detail of the Nrf2 activation of TEM by lactate is unknown. It had been reported that lactate stimulation could increase the intercellular ROS [22]. In our study, the fluorescence intensity of 2′,7′-dichlorofluorescein (DCF) showed that normal cancer cell CM significantly up-regulated intracellular ROS level of macrophage comparing with the lactate reduced ones or without cancer cell CM stimulation (Fig. 4a). Also, as Fig. 4a showed, exogenous lactate could recover the ROS level as normal cancer cell CM-induced. Above all, it confirmed that the lactate in the cancer cell CM was responsible for ROS formation in TEM. In the canonical pathways, Nrf2 activation is responsible for ROS increase [23]. In order to uncover whether Nrf2 activation in TEM relied on lactate-induced ROS or not, we inhibited the ROS formation in macrophages during cancer cell CM stimulation by NAC treatment (Fig. 4b). NAC treatment canceled the Nrf2 nuclear accumulation which appeared after cancer cell CM stimulation (Fig. 4c). Moreover, ROS inhibition diminished the macrophage phenotype remodeling (Fig. 4d-e and Additional file 1: Figure S3C-D).

### TEM activated Nrf2 of cancer cells to promote cancer cell EMT

**Fig. 5:**
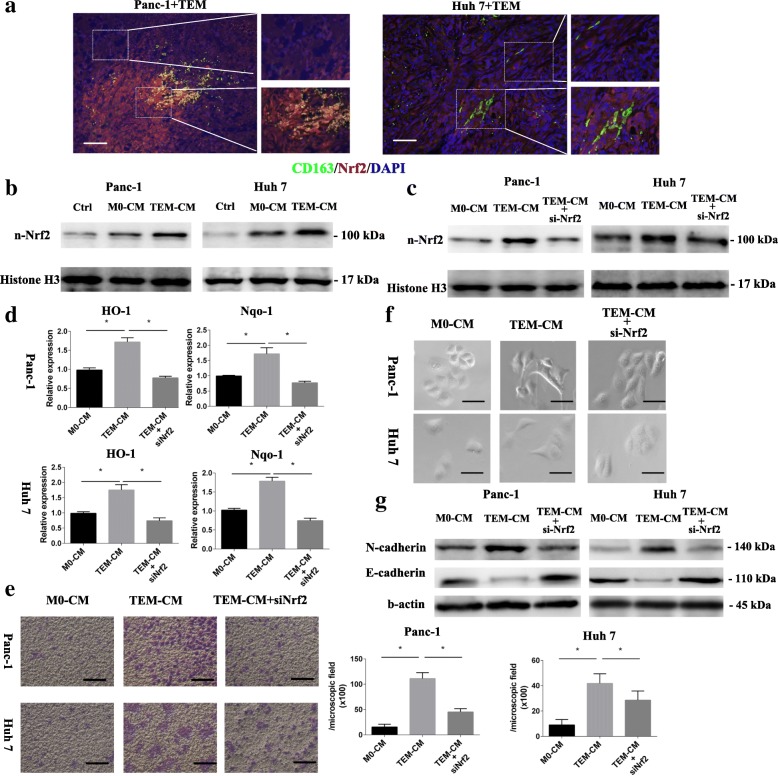
TEM activated Nrf2 of cancer cells was correlated with cancer cell EMT. **a** the tumor tissue of mice xenograft model stained with CD163 (green), Nrf2 (red) and DAPI (blue) (scale bar 100 μm) (*n* = 4). **b** nuclear Nrf2 of cancer cell was measured after stimulated with FBS-free medium (Ctrl), CM of M0 macrophages (M0-CM) or TEM (TEM-CM) by western blotting. **c** nuclear Nrf2 level of cancer cells after Nrf2 knocked down (si-Nrf2) and stimulated with TEM-CM or M0 CM were detected by western blotting. **d** the HO-1 and Nqo-1 expression of cancer cells were detected by PCR (*n* = 4). **e** the migrated cells of normal stimulated with M0 CM (M0-CM) and TEM CM (TEM-CM) and Nrf2 knocked down cancer cell stimulated with TEM CM (TEM-CM + si-Nrf2) were observed and counted (scale bar 100 μm) (*n* = 3). **f** the cancer cell morphology changes were observed after 24 h incubation with the M0 CM (M0-CM), TEM CM (TEM-CM) or Nrf2 knockdown followed TEM CM (TEM-CM + siNrf2) (scale bar 50 μm). **g** E-cadherin and N-cadherin expression of normal and Nrf2 knocked down cancer cell were detected by western blot, after 24 h incubation with the M0 CM (M0-CM), TEM CM (TEM-CM) or Nrf2 knockdown followed TEM CM (TEM-CM + siNrf2). Graphs show the data as mean ± SD. *, *P* < 0.05, compared between two groups

In the CD163 and Nrf2 double staining of mice xenograft tumor tissues, the Nrf2 nuclear translocation could be observed not only in CD163-positive cells but also in the cancer cells around the CD163 positive macrophages (Fig. 5a). In the patients’ tumor tissues, the nuclear Nrf2 positive cancer cells could also observe near the CD163 positive cells (Additional file 1: Figure S4A). In order to uncover that whether the Nrf2 activation of cancer cells correlated with CD163-positive macrophages stimulation or not, we stimulated cancer cells with CM of TEM or M0 macrophages in vitro. The higher Nrf2 nuclear accumulation of cancer cell was detected after stimulation with CM of TEM both derived from THP-1 or PBMC (Fig. 5b and Additional file 1: Figure S4B). It has been reported that Nrf2 activation could promote cancer metastasis by modulating epithelial-mesenchymal transition (EMT) [24]. Nrf2 knocked-down in cancer cells before TEM CM stimulation was performed to illustrate the relationship between TEM-induced cancer cell Nrf2 activation and EMT (Fig. 5c-d and Additional file 1: Figure. S4C-D). The migration assay showed that TEM CM significantly increased the cancer cell migration compared with M0-CM. However, Nrf2 knocked-down of cancer cell blunted the TEM migration-promoting effect (Fig. 5e and Additional file 1: Figure S4E). After TEM CM stimulation, the morphological changes of cancer cells resembling EMT were clearly observed on normal cancer cells but not on Nrf2 knocked-down ones, with cells becoming spindle-shaped and scattered compared to cobble stone-shaped (Fig. 5f and Additional file 1: Figure S4F). Moreover, the EMT related markers expression revealed that TEM promoted cancer cell EMT through Nrf2 activation, as Nrf2 knocked-down diminished the TEM modulated EMT expression (Fig. 5g and Additional file 1: Figure S4G).

### TEM derived VEGF stimulated cancer cell EMT through Nrf2 activation

**Fig. 6:**
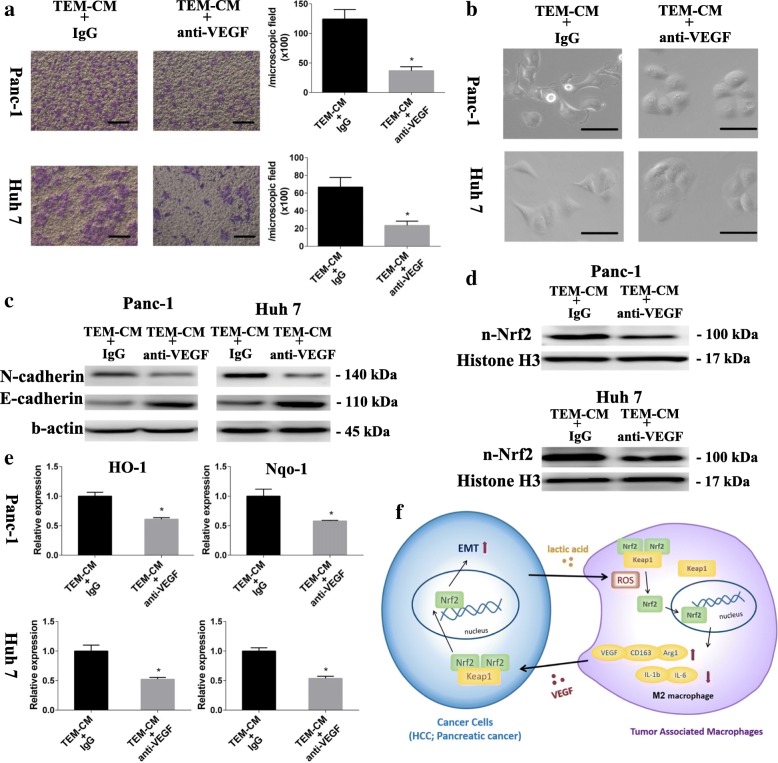
TEM derived VEGF stimulated cancer cell EMT through Nrf2 activation. **a** the migrated cancer cell stimulated with CM of TEM with (TEM-CM + anti-VEGF) or without (TEM-CM + IgG) VEGF neutralization were observed and counted (scale bar 100 μm) (*n* = 3). **b** the cancer cell morphology changes were observed after stimulated with CM of TEM with (TEM-CM + anti-VEGF) or without (TEM-CM + IgG) VEGF neutralization (scale bar 50 μm). **c** E-cadherin and N-cadherin expression of cancer cell stimulated with CM of TEM with (TEM-CM + anti-VEGF) or without (TEM-CM + IgG) VEGF neutralization were detected by western blotting. **d** nuclear Nrf2 of cancer cell was measured after stimulated with CM of TEM with (TEM-CM + anti-VEGF) or without (TEM-CM + IgG) VEGF neutralization were detected by western blotting. **e** HO-1 and Nqo-1 expression of cancer cells were detected by qPCR after stimulated with CM of TEM or VEGF neutralized (anti-VEGF) CM of TEM (*n* = 4). **f** The working model of how the cancer cells and macrophages interaction. Graphs show the data as mean ± SD. *, *P* < 0.05

Based on the previous report, VEGF could increase cancer cell proliferation, migration ability [25]. Considering that previous results showed M0 macrophages and Nrf2 knock-down TEM, both of which had lower VEGF expression than normal TEM, partly lost cancer cell migration-promoting ability, VEGF might be a candidate responsible for TEM induced cancer cell EMT. VEGF neutralization by the anti-VEGF antibody (R&D Systems, Minneapolis, MN, USA) canceled the cancer cell migration-promoting effect of TEM CM was detected (Fig. 6a and Additional file 1: Figure S5A). The morphological changes as characteristic of EMT vanished when the VEGF was neutralized (Fig 6b and Additional file 1: Figure S5B). The EMT markers expression of cancer cell also showed that VEGF reduction attenuated the EMT promoting-effect of educated macrophage (Fig. 6c and Additional file 1: Figure S5C). Also, VEGF neutralization in educated macrophage conditioned medium could not induce the Nrf2 activation and Nrf2-dependent transcription (Fig. 6d-e and Additional file 1: Figure S5D-E). Above all, it demonstrated that TEM derived VEGF stimulated cancer cell EMT through Nrf2 activation.

## Discussion

In this study, we described a new role of Nrf2, relevant for cancer-induced macrophages phenotype transformation and paracrine action of TAM on cancer cell EMT. Our study demonstrated the existence of a cross-talk between macrophages and cancer cells. Cancer cells secreted lactate, which elevated ROS in macrophages, induced macrophage M2 phenotype transformation and VEGF expression through Nrf2 mediation. On the other hand, cancer cell educated macrophages promoted cancer cell migration partially relied on the increased Nrf2 activation of cancer cell by VEGF secretion (Fig. 6f).

Peripheral blood monocytes are recruited and activated to form a wide spectrum of TAM in response to chemokines and growth factors to construct the tumor microenvironment [2]. In the tumor microenvironments, there are not only IFN-γ, TNF-a, and GM-CSF which could activate macrophages like M1 macrophages, but also IL-4, IL-10 and CSF-1 which induce M2 macrophages differentiation [26]. In our study, the cancer cells provoked M2 phenotype as demonstrated by an increase of CD163 and Arg1 and a decrease of IL-1b and IL-6 expression. The CD163^+^ or M2 macrophage, as a prognosis factor, induce the cancer progression including cancer cell proliferation, migration and invasion, and angiogenesis [5, 27, 28].

Nrf2, a key regulator for the maintenance of redox homeostasis, has been demonstrated to contribute to cell proliferation and malignant phenotypes [29, 30]. Previous decades, the role of Nrf2 in immune modulation have been recognized. As our study showed Nrf2 activation in macrophages inhibited the IL-1b and IL-6 expression, it has been demonstrated that activation of Nrf2 prevented LPS-induced upregulation of pro-inflammatory cytokines, including IL-1b and IL-6 [15]. IL-1b and IL-6 production are increased in Nrf2^−/−^ mice with dextran sulfate-induced colitis [31]. Moreover, Nrf2 could affect macrophage polarization toward the M2 phenotype through its downstream genes HO-1 [32]. Consistent with our study, Nrf2 activation in macrophage increased M2 markers including CD163 and Arg1 expression. However, some reports showed that Nrf2-deficient myeloid lineages but not Nrf2 wild-type could increase lung cancer metastasis in vivo [33, 34]. In these studies, the Nrf2 of myeloid-derived cells but not macrophage were modulated. It might be related with the pro-differentiation action of Nrf2 on myeloid lineages which affect anti-tumor immune cell formation [35].

The Warburg effect widely exists among the cancer cells provide cancer cell production for nucleotide, amino acids and lipids for proliferation [36, 37]. The Warburg effect causes increased production of lactate which could not only regulate the pH level but also modulate the inflammation of the tumor microenvironment [38, 39]. Lactate can suppress TLR-4 mediated inflammation in the liver and pancreatic tissue [40]. Also, it has been reported that lactate induces M2 like macrophages through HIF-1a mediation [41]. G protein-coupled receptor 132 is a stress-inducible transmembrane receptor that responses to lactate but not the acidic condition related to M2 macrophage transformation by Nrf2 activation [42]. Although no certain study was reported the relation between lactate and Nrf2, there is the indirect evidence in which lactate has been proved to elevate the intercellular ROS, as a Nrf2 activator, or HIF-1a which is usually reciprocal activation of Nrf2 show the clues of the relationship of lactate and Nrf2 [43].

Our results also implied that the Nrf2 over-activation in the tumor was necessarily associated with TAM promote migration through induced EMT. The association of Nrf2 and cancer malignancy has been previously shown. The previous analyses of Nrf2 expression or activation in human tumors have revealed that high Nrf2 expression or activation correlated with poor prognosis in hepatocellular carcinoma and pancreatic cancer [44, 45]. Actually, Nrf2 not only maintains cancer cell growth at oxidative stress, but also increase the cancer cell invasion potential through downregulated E-cadherin expression [13, 46].

The effect of VEGF on cancer cell migration and invasion has been reported decades ago [47]. With the anti-VEGFR treatment wildly performed in the clinical, VEGF-VEGFR induced cancer metastasis is further noticed. The association of VEGF and Nrf2 activation expression has been reported in Kweider’s study in which VEGF activated the Nrf2 in an ERK1/2-dependent manner [48]. Hypoxia due to Nrf2 activation can regulate HIF-1α/VEGF signaling pathways to modulate angiogenesis [49]. In addition, Nrf2 knockdown significantly decreased VEGF expression [50]. Our results demonstrated that TEM derived VEGF could activate Nrf2 activation in the cancer cell.

## Conclusions

In conclusion, lactate derived from cancer cells stimulate macrophages M2 phenotype transformation through Nrf2 activation. TEM secrete VEGF to promote cancer cell migration by Nrf2 induced EMT. These findings not only cover the cancer cell and macrophages interaction to contribute to tumor progression but also provides comprehensive insights into the role of Nrf2 in TAM formation and tumor metastasis.

## Additional file

Additional file 1: Supplement methods and figures. (PDF 6318 kb)

## Abbreviations

CM: Conditioned medium
DCF: 2′,7′-dichlorofluorescein
DMEM: Dulbecco’s modified eagle medium
EMT: Epithelial-mesenchymal transition
FBS: Fetal bovine serum
H2DCFDA: 2′, 7′-Dichlorofluorescin diacetate
HCC: Hepatocellular carcinoma
Nrf2: Nuclear factor (erythroid-derived 2)-like 2
PBMC: Peripheral blood monocyte
PMA: Phorbol-12-myristate-13-acetate
ROS: Reactive oxygen species
TAM: Tumor-associated macrophages
TEM: Tumor educated macrophage
