# Supplementary material for: Nrf2 activation drive macrophages polarization and cancer cell epithelial-mesenchymal transition during interaction
Source: Cell Commun Signal. 2018 Sep 4;16:54. doi: 10.1186/s12964-018-0262-x (PMC6122794; doi:10.1186/s12964-018-0262-x)
Supplement: Supplementary file 1 — Supplement methods and figures. (PDF 6318 kb) [file 12964_2018_262_MOESM1_ESM.pdf]

Supplement Methods

Human peripheral blood monocyte isolation

Human peripheral blood monocytes (PBMC) were isolated from a healthy donor. 20 ml blood was collected into EDTA containing vacutainers (170418G, Terumo, Japan) and centrifuged at 550g for 20 min. 5 ml buffy coat was collected and mixed with 4 ml Optiprep (D1556, Sigma, Japan) in a new 50 ml tube. The mixture was overlaid with 7.5 ml of a 1.078g/ml Optiprep-HBSS (1 part Optiprep and 3 part 0.5% BSA containing HBSS-). Then a 1.068 g/ml Optiperp-HBSS (1 part Optiprep and 4 part 0.5% BSA containing HBSS-) was overlaid on. On the top, there was 1 ml HBSS-. The buffy coat /Optiperp mixture was centrifuged at 600g for 25 min at room temperature. The layer which was between HBSS- and 1.068 Optiperp-HBSS was collected as monocytes. The isolated monocytes were cultured in RPMI1640 containing 10%FBS.

For the macrophage differentiation, the isolated PMBC were stimulated with 20nM M-CSF (461-ML, R&D, USA) for six days.

Flow cytometry

The detached cells were blocked the non-specific Fc-mediated by mouse serum with 15 minutes. Then the cells were incubated with the PE-conjugated anti-CD163 antibody (eBioscience, Thermo Fisher Scientific, USA) 30 minutes on ice. After three times washing, the cells were analyzed by flow cytometry. The data of flow cytometry were analyzed with FlowJo V10 (FlowJo, LLC, USA).

Immunohistochemistry

Briefly, patients' tumor tissue sections were incubated with ice-cold neutral buffer containing 10% formalin and fixed overnight at 4˚C. Fixed sections were paraffin embedded. For immunohistochemistry, 4-μm sections were deparaffinized in xylene and rehydrated sequentially in ethanol. For antigen retrieval, slides were boiled in 0.01 M sodium citrate (pH 6.0) for 30 min. Sections were blocked by incubation with 5% goat serum in PBS for 60 min. After blocking, the sections were

firstly incubated with CD163 (1:500) antibody overnight 4 °C. The sections were washed and incubated with the HRP-conjugated second antibody (Dako Japan, Tokyo, Japan) for 60 min. After washing, the sections were incubated with diamino-benzidine. Antigen retrieval and blocking as before was performed again. The sections were then incubated with the anti-Nrf2 antibody (1:250) and visualized using HistoGreen (Linaris, Dossenheim, Germany). Before mounted, nucleuses were stained with nuclear fast red (N3020, Sigma, USA).

Supplement Figure Legends

Figure S1

A, PBMC were stimulated with M-CSF (20ng/ml) for 6 days to generate macrophages (M0) then the PBMC derived macrophages were educated by cancer cell CM other 24 hours (Huh-Cm or Panc-CM). The M1/2 macrophages markers expression were detected by PCR (n=4). Graphs show the data as mean ± SD. *, *P* < 0.05, compared with M0. **B,** PBMC derived macrophages were educated with cancer cell CM for 24 hours, then Nrf2 (red) and nuclear (blue) of macrophages were stained (scale bar 100 µm). **C,** the Nrf2 level in nuclear and cytoplasm of PBMC derived macrophages were detected by western blot after 24-hour cancer cell CM education. **D**, the tumor tissue of mice xenograft stained with CD163 (green), Nrf2 (red) and DAPI (blue) (scale bar 100 µm). **E,** the tumor tissue of cancer patients were stained with CD163 (brown), Nrf2 (green) and nuclear (red) (scale bar 200 µm). The arrows showed both CD163 and nuclear Nrf2 positive cells.

Figure S2

A, HO-1 and Nqo-1 expression of macrophages were measured by PCR after stimulated with cancer cell CM (Panc-CM or Huh-CM), Nrf2 knocked-down macrophages stimulated with cancer cell CM (Panc-CM+si-Nrf2 or Huh-CM+si-Nrf2), Keap-1 knocked-down macrophages stimulated with cancer cell CM (Panc-CM+si-Keap-1 or Huh-CM+si-Keap-1), and macrophages stimulated with cancer cell CM and DEM (Panc-CM+DEM or Huh-CM+DEM) (n=4). Graphs show the data as mean ± SD. *, **P** < 0.05, compared with Panc-CM or Huh-CM. **B**, the flow cytometry analysis of CD163 stained M0 macrophages (ctrl), macrophages stimulated with cancer cell CM (Panc-CM or Huh-CM), Nrf2 knocked-down macrophages stimulated with cancer cell CM (Panc-CM+si-Nrf2 or Huh-CM+si-Nrf2), Keap-1 knocked-down macrophages stimulated with cancer cell CM (Panc-CM+si-Keap-1 or Huh-CM+si-Keap-1), and macrophages stimulated with cancer cell CM and DEM (Panc-CM+DEM or Huh-CM+DEM).

Figure S3

A, the flow cytometry analysis of CD163 stained macrophages stimulated with Panc-1 CM, lactate reduced Panc-1 CM (Panc-CM+LA-) or exogenous lactate supplement contained lactate reduced Panc-1 CM (Panc-CM+LA-/+). **B**, the flow cytometry analysis of CD163 stained macrophages stimulated with Huh 7 CM, lactate reduced Huh 7 CM (Huh-CM+LA-) or exogenous lactate supplement contained lactate reduced Huh 7 CM (Huh-CM+LA-/+). **C**, the flow cytometry analysis of CD163 stained macrophages stimulated with Panc-1 CM, or Panc-1 CM and NAC combination (Panc-CM+NAC). **D**, the flow cytometry analysis of CD163 stained macrophages stimulated with Panc-1 CM, or Panc-1 CM and NAC combination (Panc-CM+NAC).

Figure S4

A, tumor tissues were stained with CD163 (brown) and Nrf2 (green) (scale bar 200 μm).**B,** cancer cells were stimulated with FBS- free medium (Ctrl), the conditioned medium of PBMC derived macrophage (p-M0) or PBMC derived TEM (p-TEM) for 24 hours, nuclear Nrf2 levels of cancer cells were detected by western blot. **C,** cancer cells stimulated with conditioned medium of PBMC derived macrophage (p-M0) or PBMC derived TEM (p-TEM) and Nrf2-knocked-down cancer cells were stimulated with conditioned medium of PBMC derived TEM (p-TEM+si-Nrf2), nuclear Nrf2 levels of cancer cells were detected by western blot. **D**, cancer cells stimulated with conditioned medium of PBMC derived macrophage (p-M0) or PBMC derived TEM (p-TEM) and Nrf2-knocked-down cancer cells were stimulated with conditioned medium of PBMC derived TEM (p-TEM+si-Nrf2), HO-1 and Nqo-1 expression of cancer cells were measured by PCR (n=4). Graphs show the data as mean ± SD. *, $P < 0.05$, compared between two groups. **E,** cancer cells stimulated with conditioned medium of PBMC derived macrophage (p-M0) or PBMC derived TEM (p-TEM) and Nrf2-knockde down cancer cells were stimulated with conditioned medium of PBMC derived TEM (p-TEM+si-Nrf2), the migrated cancer cell were observed and counted (scale bar 200 μm) (n=3). Graphs show the data as mean ± SD. *, $P < 0.05$, compared between two groups. **F,** cancer cells stimulated with conditioned medium of PBMC derived macrophage (p-M0) or PBMC derived TEM (p-TEM) and Nrf2-knocked-down cancer cells were stimulated with conditioned medium of PBMC derived TEM (p-TEM+si-Nrf2), the morphology of cancer cells was observed (scale bar 50 μm) (n=3). **G,** cancer cells of which Nrf2 were knocked down or not were stimulated with conditioned medium of PBMC derived TEM, E-cadherin and N-cadherin expression of cancer cells were detected by western blot.

Figure S5

A, conditioned medium of PBMC derived TEM in which the VEGF was neutralized (p-TEM-CM+anti-VEGF) or not (p-TEM-CM+IgG) were used to stimulate cancer cells during migration assay, the migrated cancer cells were observed and counted (scale bar 100 μm) (n=3). Graphs show the data as mean ± SD. *, $P < 0.05$, compared between two groups. **B,** the morphology of cancer cells which stimulated with VEGF neutralized conditioned medium of PBMC derived TEM (scale bar 50 μm). **C,** the E-cadherin and N-cadherin expression of cancer cells which stimulated with VEGF neutralized conditioned medium of PBMC derived TEM were detected by western blot. **D,** the nuclear Nrf2 levels of cancer cells which stimulated with VEGF neutralized conditioned medium of PBMC derived TEM or not were evaluated by western blot. **E,** HO-1 and Nqo-1 expression of cancer cells which stimulated with VEGF neutralized conditioned medium of PBMC derived TEM were assessed by PCR (n=4). Graphs show the data as mean ± SD. *, $P < 0.05$, compared between two groups.

Figure S1

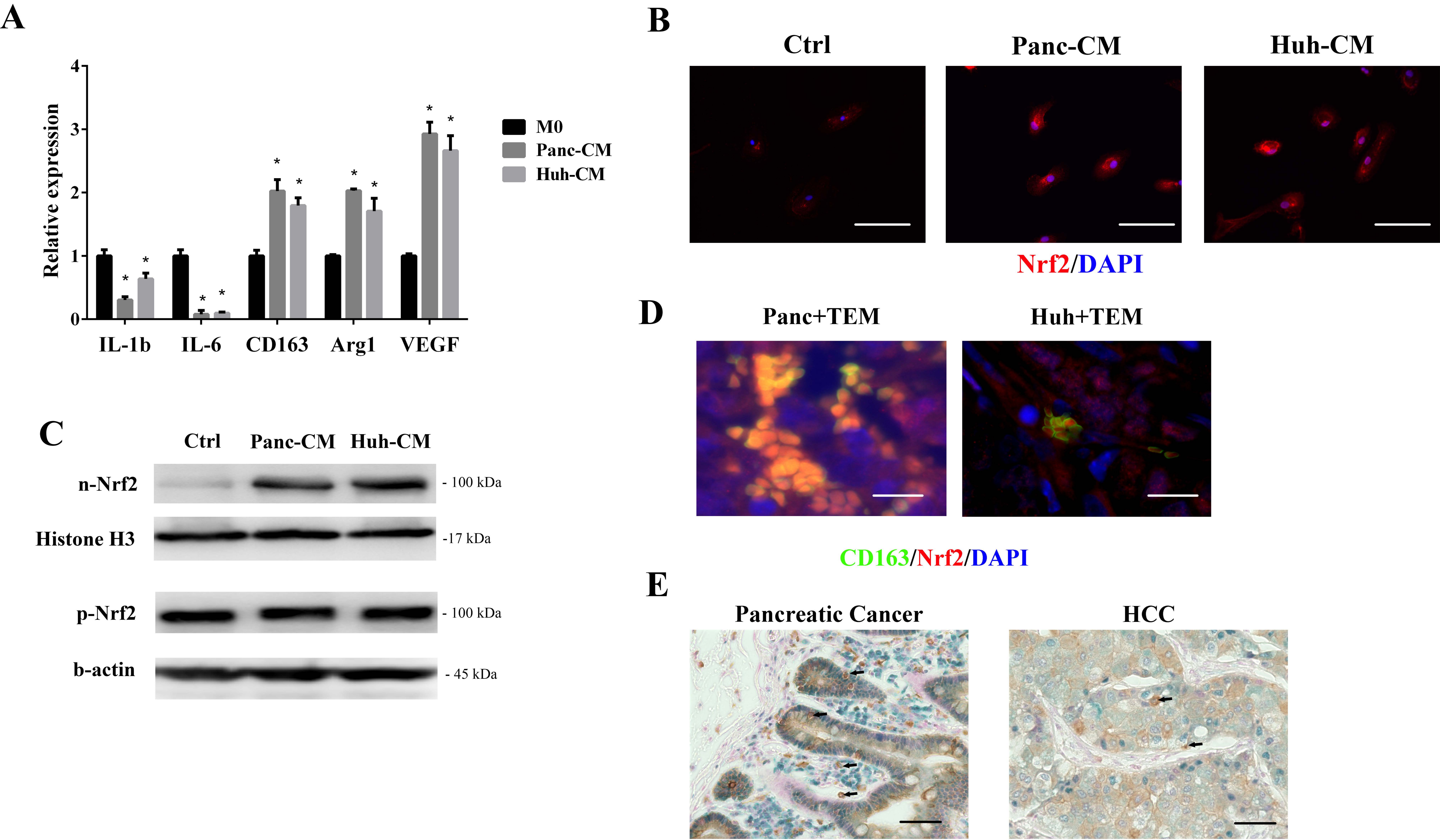
A
Relative expression
0
1
2
3
4
IL-1b
IL-6
CD163
Arg1
VEGF
M0
Panc-CM
Huh-CM
B
Ctrl
Panc-CM
Huh-CM
Nrf2/DAPI
C
Ctrl
Panc-CM
Huh-CM
n-Nrf2
- 100 kDa
Histone H3
-17 kDa
p-Nrf2
- 100 kDa
b-actin
- 45 kDa
D
Panc+TEM
Huh+TEM
CD163/Nrf2/DAPI
E
Pancreatic Cancer
HCC

Figure S2

A

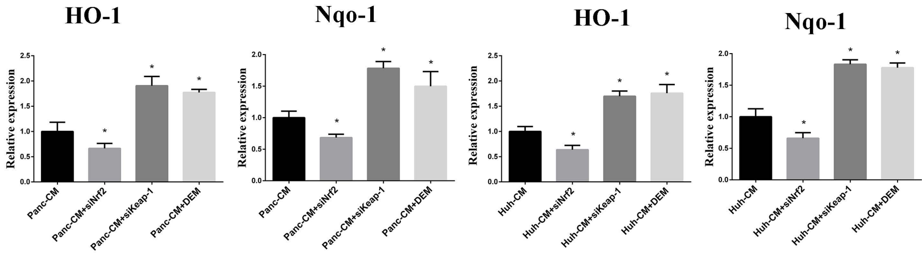

B

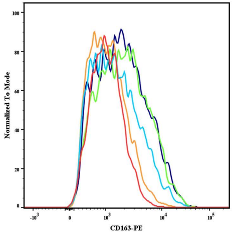

	Sample Name	Mean of PE
	Ctrl	1363
	Panc-CM	2468
	Panc-CM+si-Nrf2	1604
	Panc-CM+si-Keap-1	3404
	Panc-CM+DEM	3389

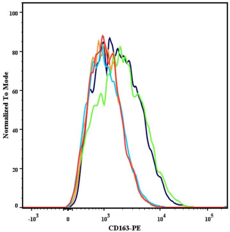

	Sample Name	Mean of PE
	Ctrl	1363
	Huh-CM	1652
	Huh-CM+si-Nrf2	1395
	Huh-CM+si-Keap-1	3125
	Huh-CM+DEM	2760

Figure S3

A

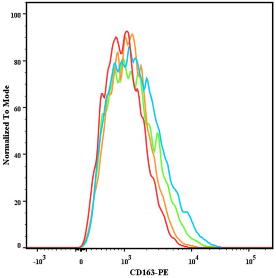

	Sample Name	Mean of PE
	Ctrl	1271
	Panc-CM	2284
	Panc-CM+LA-	1513
	Panc-CM+LA-/+	1903

B

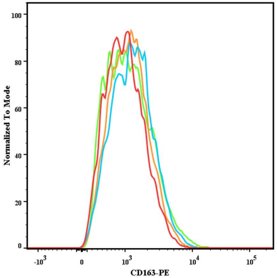

	Sample Name	Mean of PE
	Ctrl	1271
	Huh-CM	1635
	Huh-CM+LA-	1455
	Huh-CM+LA-/+	1606

C

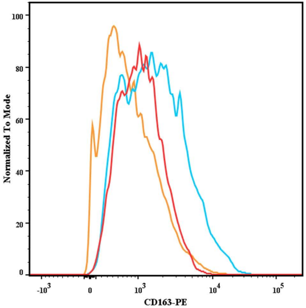

	Sample Name	Mean of PE
	Ctrl	1398
	Panc-CM	2598
	Panc-CM+NAC	1128

D

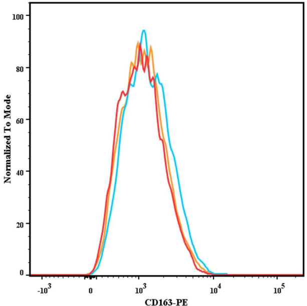

	Sample Name	Mean of PE
	Ctrl	1398
	Huh-CM	1707
	Huh-CM+NAC	1487

Figure S4

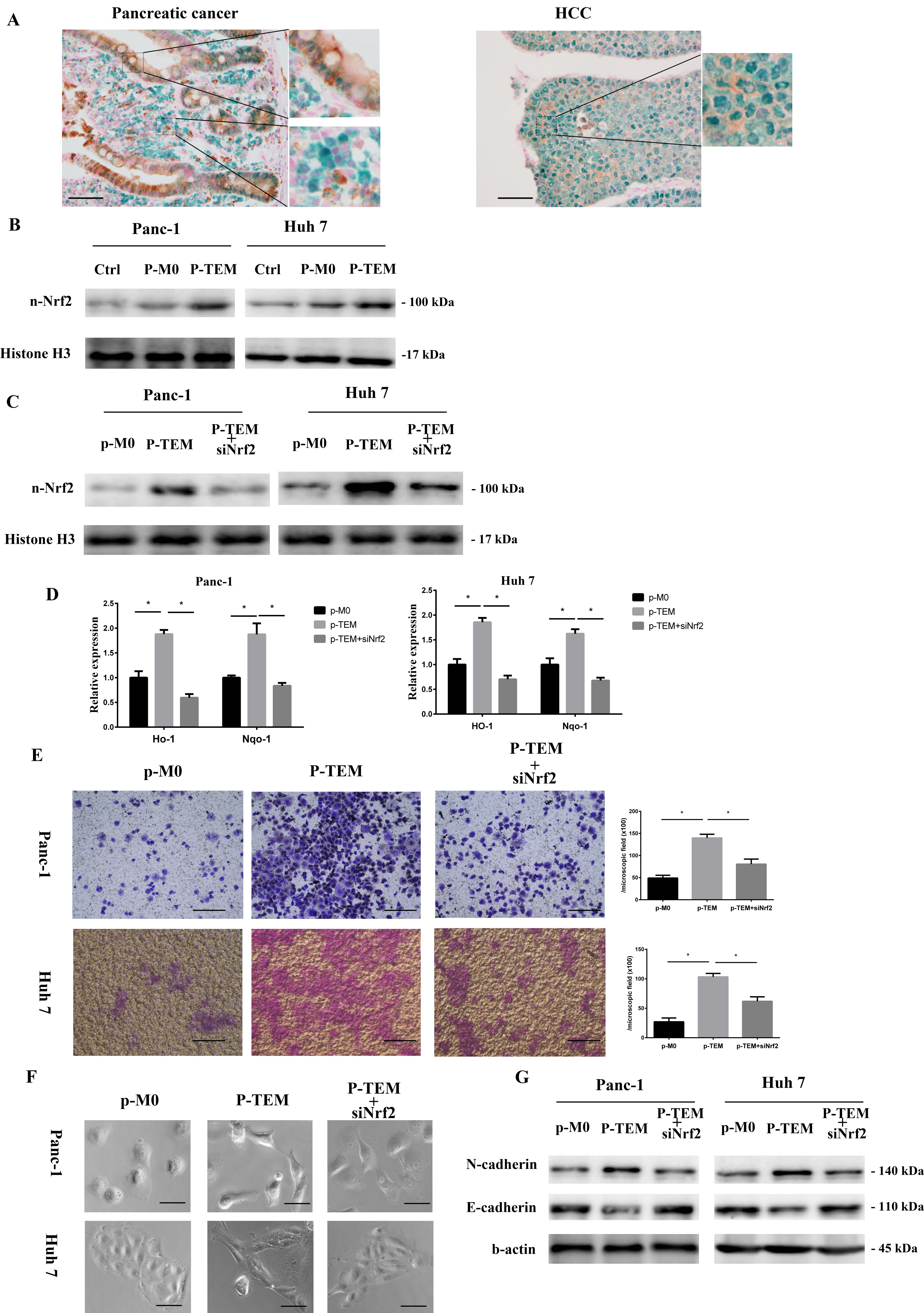

A
Pancreatic cancer
HCC
B
Panc-1
Huh 7
Ctrl P-M0 P-TEM Ctrl P-M0 P-TEM
n-Nrf2
- 100 kDa
Histone H3
-17 kDa
C
Panc-1
Huh 7
p-M0 P-TEM P-TEM + siNrf2 p-M0 P-TEM P-TEM + siNrf2
n-Nrf2
- 100 kDa
Histone H3
- 17 kDa
D
Panc-1
Huh 7
Relative expression
Ho-1
Nqo-1
HO-1
p-M0
p-TEM
p-TEM+siNrf2
E
p-M0
P-TEM
P-TEM + siNrf2
Panc-1
Huh 7
/microscopic field (x100)
F
p-M0
P-TEM
P-TEM + siNrf2
Panc-1
Huh 7
G
Panc-1
Huh 7
p-M0 P-TEM P-TEM + siNrf2 p-M0 P-TEM P-TEM + siNrf2
N-cadherin
- 140 kDa
E-cadherin
- 110 kDa
b-actin
- 45 kDa

Figure S5

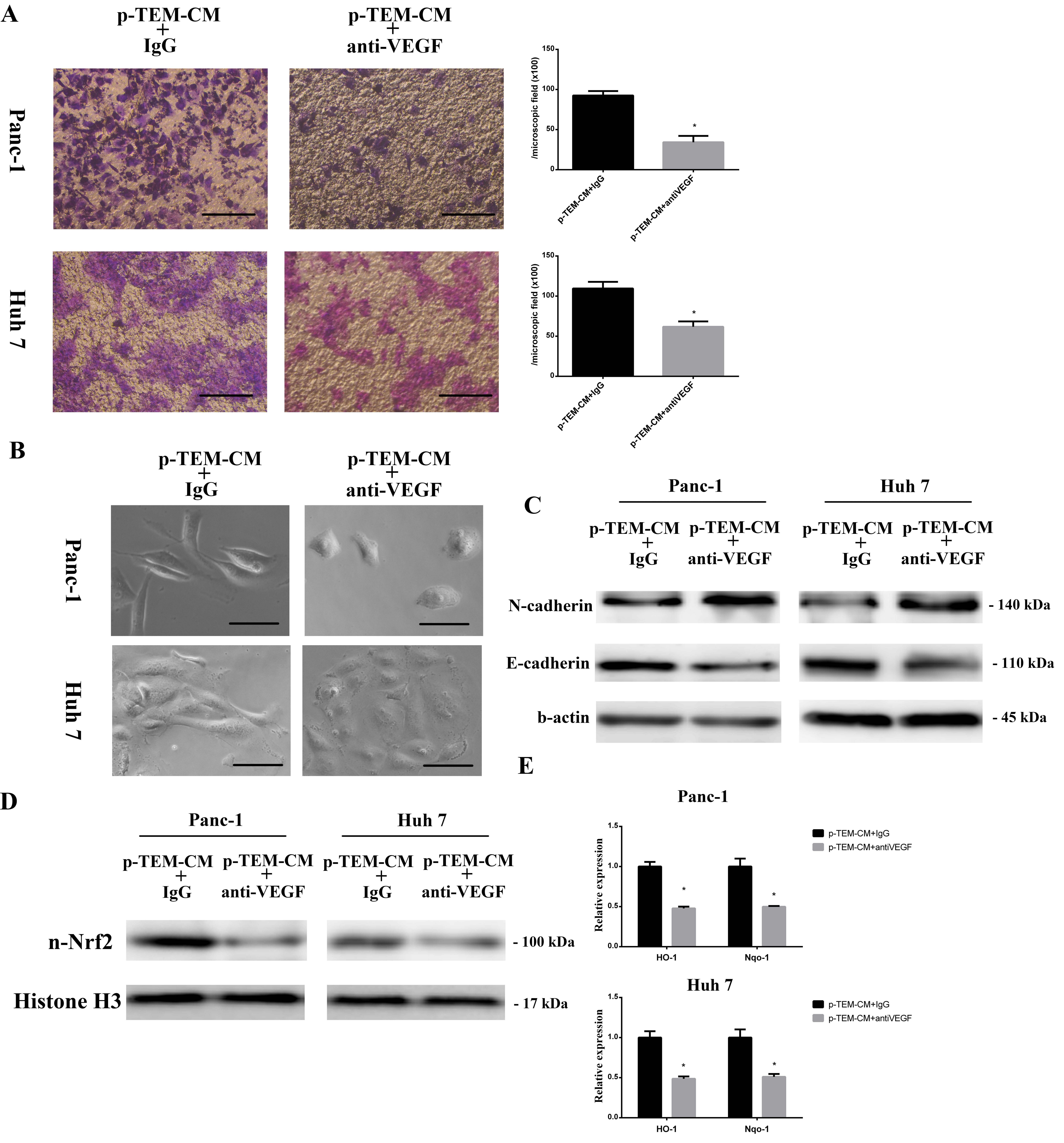
A
p-TEM-CM + IgG
p-TEM-CM + anti-VEGF
Panc-1
Huh 7
/microscopic field (x100)
150
100
50
0
*
p-TEM-CM+IgG
p-TEM-CM+antiVEGF
B
p-TEM-CM + IgG
p-TEM-CM + anti-VEGF
Panc-1
Huh 7
C
Panc-1
Huh 7
p-TEM-CM + IgG
p-TEM-CM + anti-VEGF
N-cadherin
E-cadherin
b-actin
- 140 kDa
- 110 kDa
- 45 kDa
D
Panc-1
Huh 7
p-TEM-CM + IgG
p-TEM-CM + anti-VEGF
n-Nrf2
Histone H3
- 100 kDa
- 17 kDa
E
Panc-1
Huh 7
Relative expression
1.5
1.0
0.5
0.0
HO-1
Nqo-1
p-TEM-CM+IgG
p-TEM-CM+antiVEGF
